# The Potential of Micellar Media in the Synthesis of DNA‐Encoded Libraries

**DOI:** 10.1002/chem.202103967

**Published:** 2022-02-14

**Authors:** Réka Adamik, Balázs Buchholcz, Ferenc Darvas, Gellért Sipos, Zoltán Novák

**Affiliations:** ^1^ ELTE “Lendület” Catalysis and Organic Synthesis Research Group Institute of Chemistry Eötvös Loránd University Pázmány Péter stny. 1/A 1117 Budapest Hungary; ^2^ Innostudio Inc. Záhony u. 7 1031 Budapest Hungary; ^3^ ComInnex Inc. Záhony u. 7 1031 Budapest Hungary

**Keywords:** catalysis, DNA, micelles, transition metals, water

## Abstract

DNA‐encoded library (DEL) technology has become widely used in drug discovery research. The construction of DELs requires robust organic transformations that proceed in aqueous media under mild conditions. Unfortunately, the application of water as reaction medium for organic synthesis is not evident due to the generally limited solubility of organic reagents. However, the use of surfactants can offer a solution to this issue. Oil‐in‐water microemulsions formed by surfactant micelles are able to localize hydrophobic reagents inside them, resulting in high local concentrations of the organic substances in an otherwise poorly solvated environment. This review provides a conceptual and critical summary of micellar synthesis possibilities that are well suited to DEL synthesis. Existing examples of micellar DEL approaches, together with a selection of micellar organic transformations fundamentally suitable for DEL are discussed.

## Graphical abstract

**DNA‐encoded libraries (DELs)** have become a widely used technology in drug discovery. The use of micelle‐forming surfactants provides new possibilities for synthetic organic chemistry and could be a promising technique for DNA‐compatible chemistry as well. This review provides a summary of existing examples of micellar DEL approaches, together with a selection of micellar organic transformations fundamentally suitable for DEL synthesis.

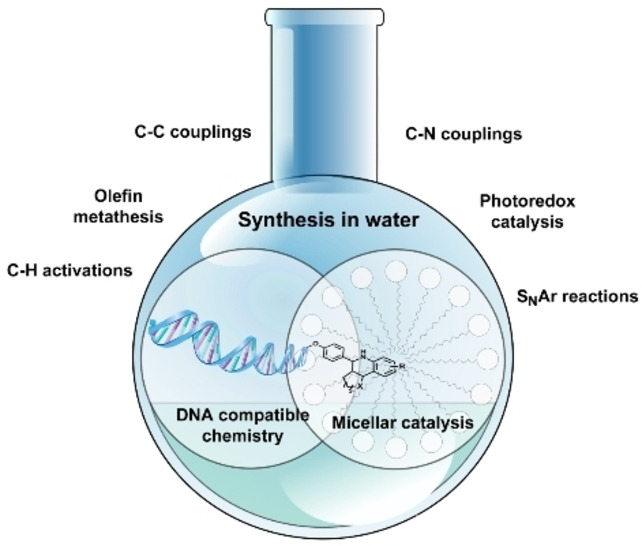

## 1 Introduction

The first proposal of the concept of encoding a chemical library with sequenced nucleotide tags was published in 1992 by Brenner and Lerner.^[1]^ In the last decades, this idea has evolved to a frequently applied hit‐finding technology for early‐phase drug discovery.^[2]^ These days, DNA‐encoded library (DEL) technology offers a complementary and powerful alternative to traditional high‐throughput screening (HTS) techniques.^[3]^

During DEL synthesis, huge libraries of molecules, potentially containing many billions of individual entities covalently attached to DNA tags can be created by using water‐based combinatorial chemistry approaches. The success of DEL ultimately relies on the quality and diversity of the libraries. However, the number of available DEL‐compatible organic reaction methodologies is limited, and their development can be quite challenging. Most importantly, such reactions need to proceed under DNA‐compatible reaction conditions and should provide high yields over a broad range (hundreds) of substrates.

Micelle‐aided organic transformations has become widely used both in academical research and industrial applications in the last few years,^[4]^ as the use of surfactants allows the implementation of several organic synthetic methods in aqueous media under mild conditions. Not surprisingly, micellar conditions have found application in DEL chemistry as well.

Recent reviews on micellar catalysis as well as DEL synthesis are available. However, we are not aware of contributions analyzing the interdisciplinary field of micellar DEL chemistry. This review aims to provide a conceptual and critical summary of micellar synthesis possibilities that are well‐suited for DEL synthesis. We discuss existing examples on micellar DEL approaches and present a selection of organic and catalytic transformations performed under micellar conditions in water, which could be suitable for the synthesis of DNA tagged molecules and DNA‐encoded libraries, thus could provide new opportunities for future synthetic technologies for DEL chemistry.

### 1.1 DNA‐encoded libraries, synthesis and application

**Scheme 1:**
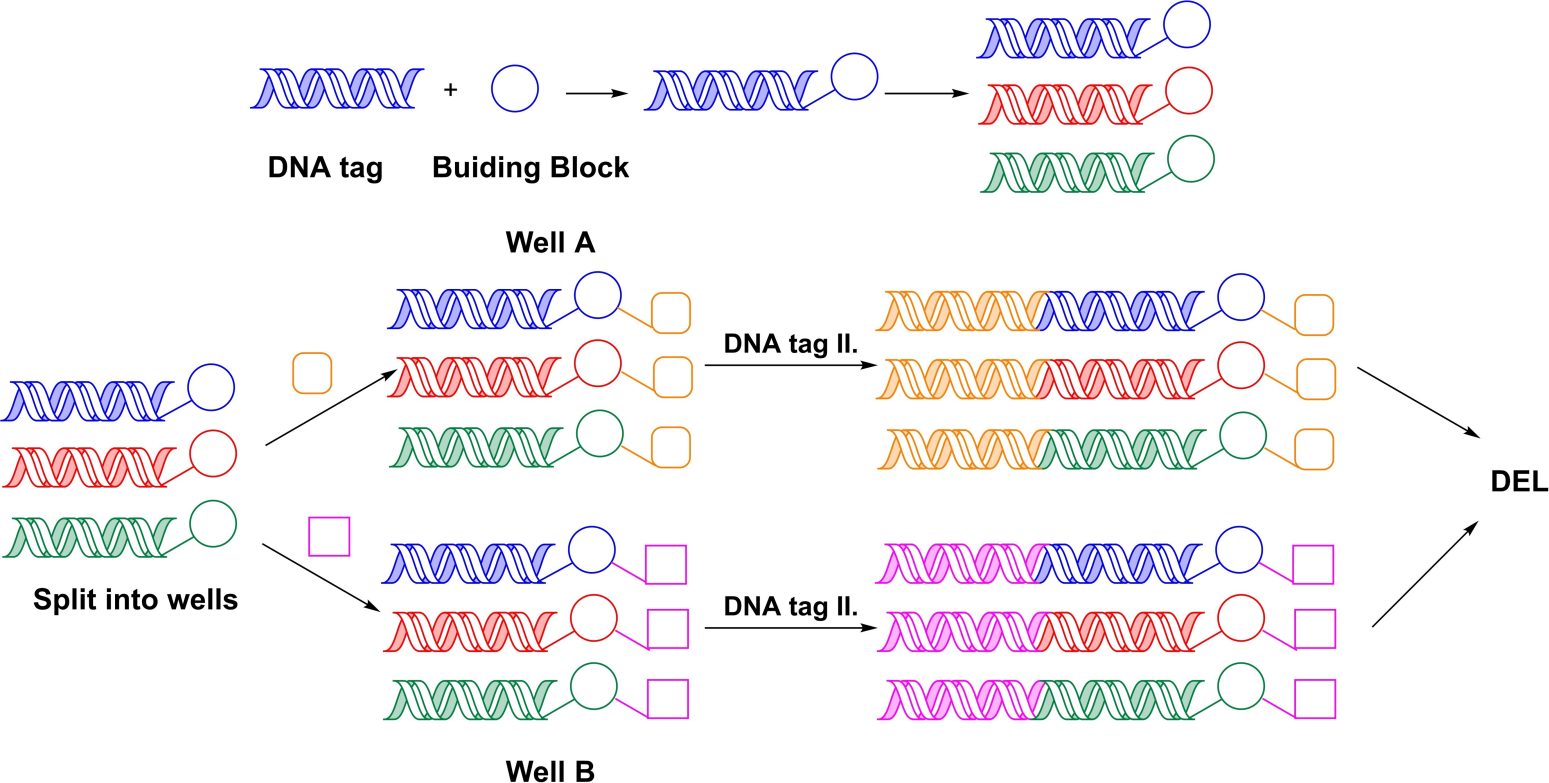
Construction of a DNA‐encoded library by using combinatorial split‐and‐pool chemistry.

**Scheme 2:**
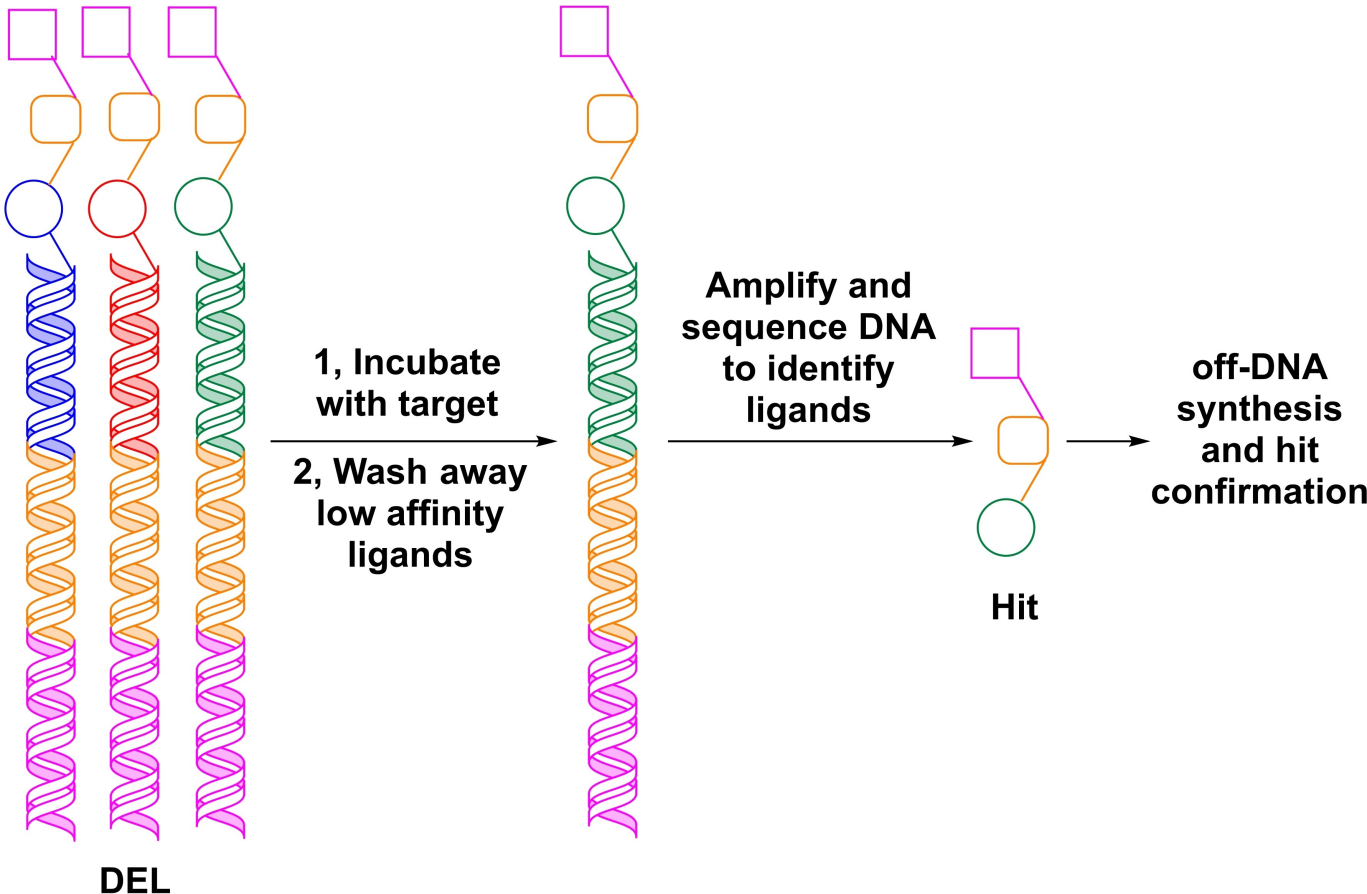
Use of DNA‐encoded libraries in drug discovery.

There are several techniques to construct a DNA‐encoded library, however the most widely applied method is split‐and‐pool combinatorial chemistry (Scheme 1). In a typical process, a chemically modified DNA starting material, commonly called the headpiece, is used as the starting point for DEL synthesis. The headpiece has functionality for both small molecule synthetic organic chemistry and DNA ligation. On one end the small molecule entity is built, while on the other end DNA tags are added. Addition of each building block is accompanied by a ligation experiment. The experimental procedure is designed so that the DNA tags can function as barcodes for the building blocks added. DELs are normally built through 2–4 cycles and contain up to 10^8^ members. These libraries can be stored in simple Eppendorf tubes and might be used in hundreds of affinity screening experiments (Scheme 2). Once a screening experiment is performed the hits are decoded using PCR‐amplification followed by sequencing. Finally, the process ends with off‐DNA confirmation of the hits.

Reaction development for DEL synthesis can be a challenging task due to several factors.^[5]^ To be practically useful a reaction must be robust and high yielding. Although there is no consensus on yield requirements, a >70 % threshold for HPLC yield seems to be generally acceptable.[^5a^, ^5b^] Furthermore, the reaction should proceed with high selectivity so high fidelity hits can be obtained during affinity screening. From a chemical standpoint this translates to several requirements. In the one hand, oligonucleotides have very low solubility in organic solvents, hence DEL synthesis generally needs to be conducted in aqueous environment. On the other hand, most of the organic reagents have low solubility in water. To tackle these contradicting requirements aprotic organic co‐solvents (e. g., DMA, DMF) are normally used. Furthermore, the DNA substrate is used at low concentration (∼1 mM), and all other reagents are added in large excess (up to thousands of equivalents). This strategy is economically viable as the DNA material is used on nmol scale, meaning that organic reagents are generally required in less than milligram amounts.

Another important aspect is that DNA integrity must be sustained during library synthesis.^[6]^ Degradation or modification of the DNA would lead to decline in ligation efficiency and/or amplifiability, overall resulting in a negative effect on the decoding of the library. Therefore, acidic conditions, high temperatures, UV light and certain reagents (e. g., oxidizing agents, radical initiators) which would corrupt the genetic information of the DNA should be avoided.

**Scheme 3:**
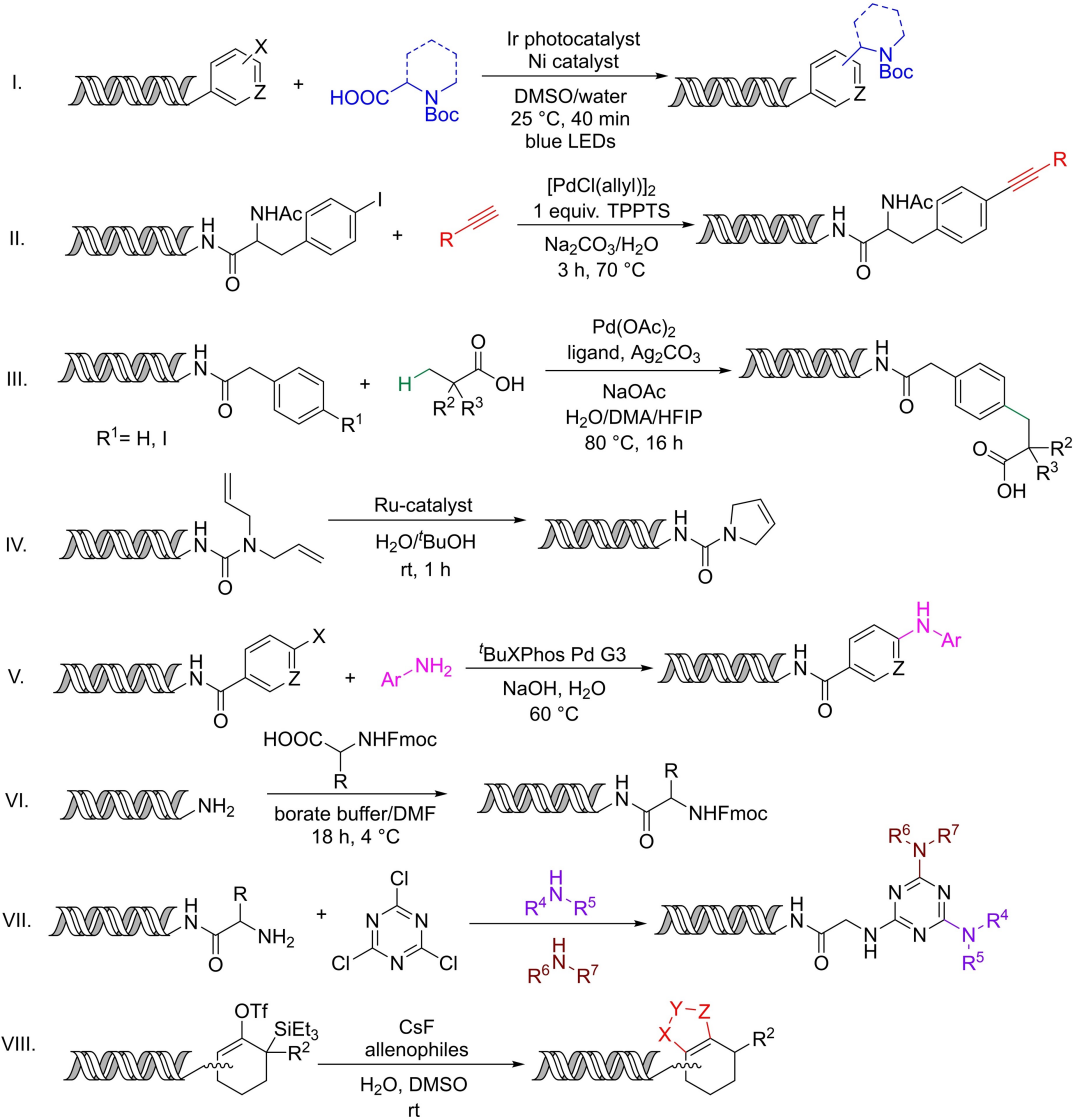
Examples of DNA compatible organic transformations: photoredox catalysis, C−C cross‐coupling, C−H activation, olefin metathesis, Buchwald–Hartwig reaction, amide formation, aromatic nucleophilic substitution and Diels‐Alder reaction.

Nonetheless, several DNA compatible conditions have been developed over the years[^5^, ^7^] such as amide bond formations,^[8]^ Diels‐Alder reactions,^[9]^ nucleophilic aromatic substitutions,^[10]^ cross‐coupling reactions^[11]^ and complex synthetic methods like photoredox catalysis,^[12]^ C−H activations,^[13]^ and olefin metathesis^[14]^ (Scheme 3). It is worth noting that most of the novel chemistries have not been shown in actual DEL production to date.

### 1.2 Micellar chemistry

Historically, organic transformations are carried out using organic solvents based on the principle of “like dissolves like”. However, in the last few decades increasing efforts have been dedicated to the elimination of negative environmental and health impact of organic solvents. Hence, the need for alternative reaction media became apparent. Water would be an obvious choice since it is reasonably low‐cost, nontoxic, nonflammable, safe and easily accessible. Nonetheless, with the use of water the problem of solubilizing hydrophobic organic reagents and catalysts needs to be addressed. Surfactants can offer a solution to the issue of solubility.^[15]^

Surfactant molecules applied over the critical micelle concentration (CMC) form nanometer sized micelles of variable sizes in the solution.^[16]^ These micelles can solubilize hydrophobic substances and act as nanoreactors. Micellar chemistry not only enables to overcome the low aqueous solubility of many organic substrates but might also offer a protective environment. The micelles provide a dynamic environment, in which there is a continuous exchange of the components (reagents, substrates, catalysts and products) between different micelles. The extent of solubilization depends on the temperature, the structure of the surfactant and the substrate, and the surfactant concentration as well. The degree of solubilization can be enhanced by adding controlled amount of a co‐solvent (typically acetone, toluene, THF or ethyl acetate).^[17]^

**Scheme 4:**
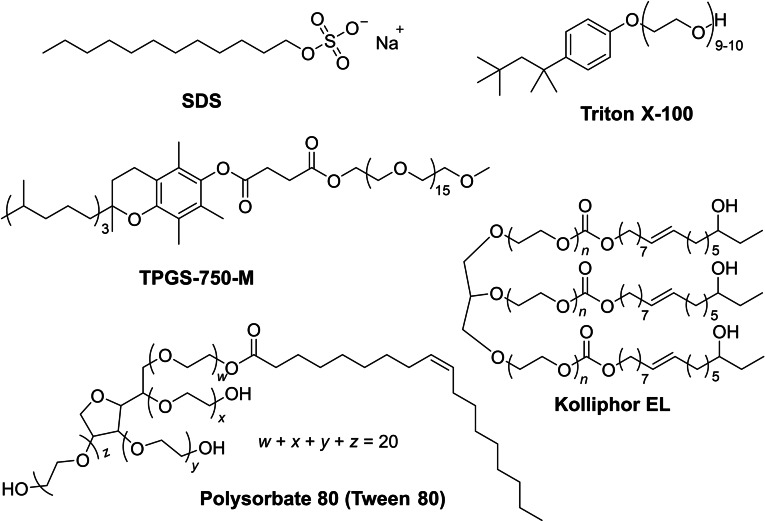
The most frequently used surfactants in micellar catalysis.

Generally, surfactants can be categorized into four categories: cationic, anionic, amphoteric and non‐ionic surfactants. Several non‐ionic surfactants are widely used in the pharmaceutical and food industries. Polysorbate 80 (also known as Tween 80) is an emulsifier applied in cosmetics and food products, and Triton X‐100 is a commonly used detergent in biological laboratories. There are a number of surfactants available on the market, ranging from traditional ones such as SDS, to new designer surfactants such as the Lipshutz group's TPGS‐750‐M (Scheme 4).^[18]^

Usually, 2–3 wt% surfactants are applied during micellar catalysis. This value is way above the CMC, especially for non‐ionic surfactants. More than 3 wt% surfactant concentration is often detrimental because it reduces the effective concentration, and as a result decreases the reaction rate as well as the exchange process, and at the same time the mixture will exhibit increased viscosity.

Micellar media as an alternative to organic solvents already have an established spot in the pharmaceutical chemistry.^[19]^ Several examples can be found in the literature for the synthesis of known drug structures under micellar conditions.^[20]^ In addition, in partnership with lead researchers of the field, Novartis is actively engaged in research projects investigating the application of micellar media for the large‐scale synthesis of APIs.^[21]^ Moreover, micellar conditions are suitable for transformations using enzymes allowing combining bio‐catalysis with chemo‐catalysis for the preparation of optically active compounds.^[22]^

## 2 Micellar Synthesis of DNA‐Tagged Molecules

**Scheme 5:**
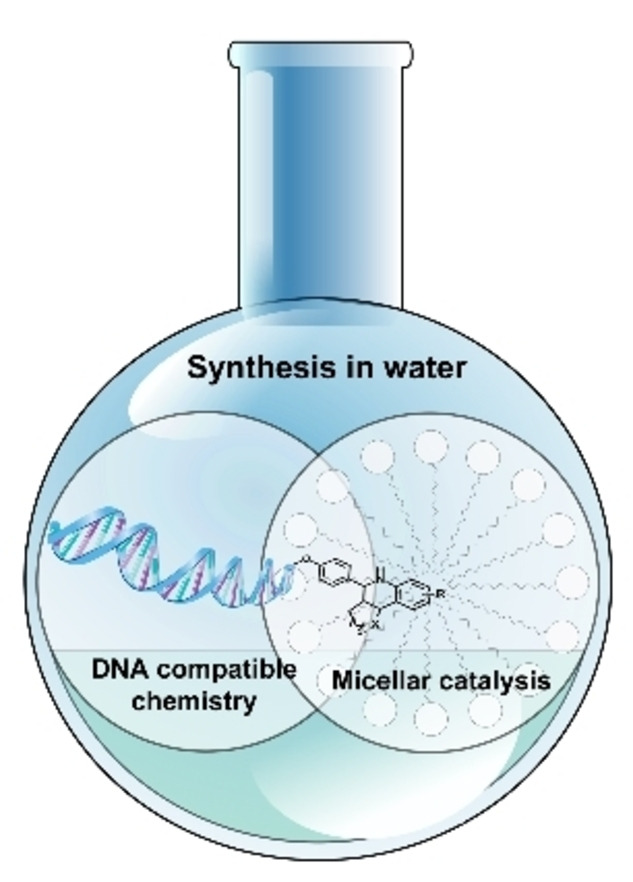
Merging micellar chemistry with DNA compatible chemistry for the construction of DNA‐encoded libraries.

Micellar conditions can offer alternative reaction media for DEL chemistry. Accelerated reactions rates can be achieved under mild conditions in micellar aqueous systems since organic reagents can move inside the micelles leading to high local concentrations in an otherwise poorly solvated environment.^[15b]^ Micellar reactions usually proceed under mild conditions (<60 °C) and offer the compartmentalization of different reagents based on their hydrophilic/phobic nature in aqueous media. These properties seem to render micellar chemistry ideal for DEL applications (Scheme 5). Nevertheless, micellar nanoreactors do not separate the hydrophilic DNA tags from the reaction site, as the DNA can tightly interact with the surfactants. Therefore, to avoid corrupting the genetic information of the DNA tags, the reaction conditions for encoded micellar chemistry still need to be chosen wisely, just as in the case of homogeneous reactions. There are only a few instances for micelle aided transformations of DNA‐tagged molecules up to date. Below we present promising examples of micellar DNA‐encoded chemistry.

### 2.1 Palladium‐catalyzed transformations

From the broad scope of palladium‐catalyzed coupling reactions Suzuki‐Miyaura couplings have been relatively well explored for DEL synthesis.^[23]^ Nevertheless, some limitations remain: a) excellent yields have been reported for phenyl iodides, but challenging substrate combinations (e. g., heteroaryl halides and/or heteroaryl boronic acids) are often low yielding; b) the demanding reaction conditions can destroy 70 % of amplifiable DNA.^[24]^

**Scheme 6:**
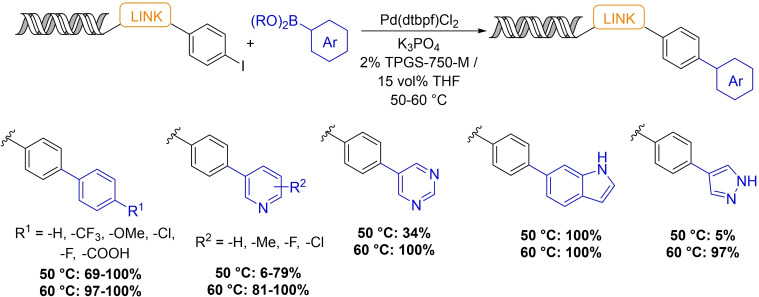
Suzuki‐coupling of DNA‐tagged molecules in aqueous TPGS‐750‐M solution.

Waring and co‐workers published a high‐fidelity method for micellar Suzuki‐coupling on DNA‐tagged substrates (Scheme 6).^[25]^ The method uses Pd(dtbpf)Cl_2_ catalyst, K_3_PO_4_ base and commercially available 2 wt% TPGS‐750‐M surfactant solution with 15 vol % THF as the solvent system. Optimization was performed in several iterations. Factorial experimental design was applied in order to achieve conditions suitable for a diverse range of boronic acids. High yields were achieved for aryl iodides with no detectable DNA damage. The authors showed that the application of TPGS‐750‐M surfactant accelerates the rate of the reaction and the addition of THF is necessary to hinder dehalogenation of the starting material.

The optimized method was utilized in a prototypical DEL synthesis. A 2D, 36‐member library was prepared. The addition of 6 aryl halide‐bearing carboxylic acids to an amino‐tagged DNA headpiece was followed by Suzuki‐Miyaura coupling with aryl boronates. At both stages a coding sequence unique to each building block was ligated followed by a closing sequence. As for quality control of the library, PCR amplification followed by gel electrophoresis and next generation sequencing was carried out. Gel electrophoresis indicated that the DNA remained intact, and all expected coding amplicons were identified after sequencing.

This research was extended to study the coupling efficiency of structurally more diverse aryl halides.^[26]^ The reaction was found to be compatible with a diverse array of aryl bromides and iodides, and the transformation tolerates functional groups that would be necessary to create a drug‐like DEL (i. e., strong hydrogen bond donors and acceptors, carboxylic acids, amines and a variety of heterocycles).

**Scheme 7:**
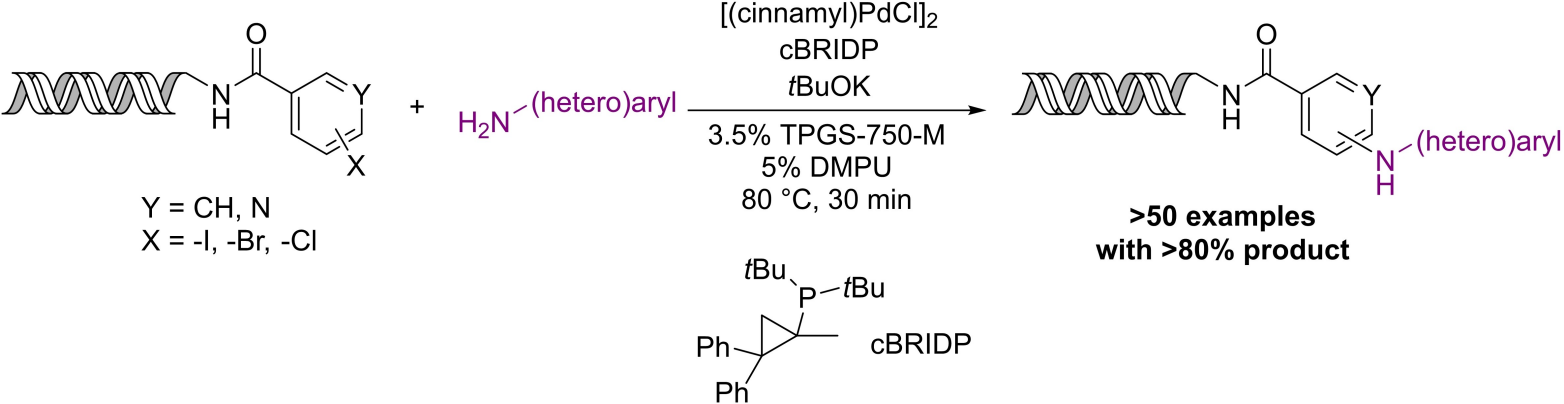
DNA‐compatible Buchwald–Hartwig amination developed by the Waring research group.

Buchwald–Hartwig and Ullmann‐type reactions are represented in the DEL literature, however the performance of these transformations is usually suboptimal, and greater than 70 % conversion was only achieved for a limited number of substrates.[^27^, ^50^] Very recently, Waring and co‐workers developed a micelle‐aided Buchwald–Hartwig amination reaction using [(cinnamyl)PdCl]_2_ precatalyst, cBRIDP ligand and *t*BuOK base (Scheme 7).^[28]^

Initial experiments revealed that the addition of a cosolvent increased the amount of product formed and suppressed dehalogenation of the starting material (similarly to their Suzuki‐coupling). The best results were achieved with 5 % DMPU. Overall, more than 50 substrate combinations were tested and most of them afforded high product percentage. Despite the excellent chemical efficiency, the requirement for large excess of the amine coupling partner (2400 equiv) might prove prohibitive in library production.

### 2.2 Amide bond formation

**Scheme 8:**
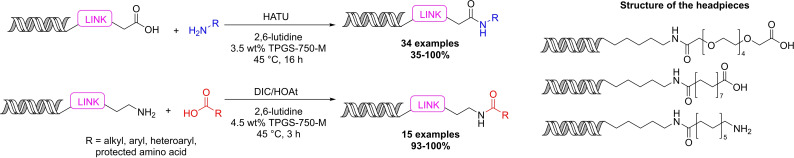
Micelle‐aided formation of amide bonds on DNA tagged molecules, and the structures of the applied headpieces.

Amide bond formations are amongst the most widely studied transformations in the DEL area.^[29]^ Efficient methods are available when a DNA‐conjugated amine is to be coupled with a carboxylic acid (C‐to‐N direction). However, the number of reports on amidations of DNA‐conjugated carboxylates (*N*‐to‐*C* direction) are scarce.^[30]^ Waring et al. investigated both *C*‐to‐*N* and *N*‐to‐*C* amidations under micellar conditions.^[31]^ Initial investigations showed that the structure of the linker between the oligonucleotide and organic handles greatly affects the outcome. While the reaction with the commonly employed PEG‐4 linker performed poorly, a more hydrophobic hexadecanoic acid linker provided promising results in the *N*‐to‐*C* coupling (Scheme 8).

Methodology development for both coupling directions involved the screening of different coupling reagents (e. g., HATU, DIC/HOAt, DMT‐MM) and factorial experimental design for the optimization of temperature, surfactant strength and base concentration. Notably, the *N*‐to‐*C* coupling utilized DIC/HOAt as coupling reagents and proceeded with high efficiency through a broad scope of substrates.

Transmission electron microscopy (TEM) images of the mixture of amino‐C11‐hexylamido DNA conjugate and TPGS‐750‐M solution showed the formation of larger conglomerates of approximately 200 nm in size. Because the sample containing only the surfactant had only about 50 nm sized micelles, the authors reasoned that the TEM images indicate that the DNA conjugates interact with the micelle forming agent and overall suggest that the micelles alter their size and shape as a result.

To demonstrate the applicability of the reaction to library synthesis, a fully‐encoded compound was also prepared on the analogy of which would be used in a library synthesis. Both building block couplings and the ligation steps occurred efficiently throughout the three‐cycle synthesis. PCR amplification and next generation sequencing of the final product confirmed the integrity of the coding DNA sequence.

### 2.3 Protic acid‐catalyzed reactions

**Scheme 9:**
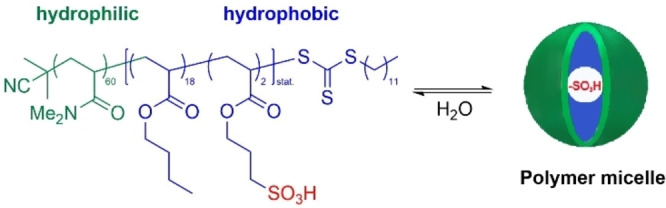
The design of the acidic functionalized block copolymer and its formation into micelles in water. Graphic adapted with permission from ref. [32a]. Copyright: 2019, American Chemical Society.

The development of DNA compatible protic acid catalyzed reactions represents a formidable challenge because pH values below 3 can lead to depurination. Weberskirch, Brunschweiger and co‐workers prepared amphiphilic block copolymers functionalized with sulfonic acid motifs.^[32]^ The block polymers formed micelles in aqueous conditions at micromolar concentration (Scheme 9).

Copolymer micelle–DNA interactions were studied by SAXS, circular dichroism, UV/VIS absorption measurements and fluorescence‐quenching experiments. Experimental data indicated strong interaction between the micelles (formed by the block copolymers) and DNA conjugates in water. It was also shown that neither the form nor the size of the micelles was significantly affected by the presence of the DNA conjugates. Fluorescence quenching experiments, as well as circular dichroism spectroscopy showed that the water soluble DNA‐oligomer‐conjugates accumulated very effectively in the micellar compartments as an essential prerequisite for carrying out a DNA‐encoded reaction in the presence of polymer micelles.

The utility of sulfonic acid functionalized copolymers was demonstrated in Povarov, Gröbke‐Blackburn‐Bienaymé, and Biginelli reactions, and also for Boc protecting group removal. Oxidation of DNA conjugated benzyl alcohol to aldehyde was performed by a micellar Cu/bipyridine/TEMPO catalyst system in which the bipyridine unit was incorporated into the amphiphilic block copolymer.

**Scheme 10:**
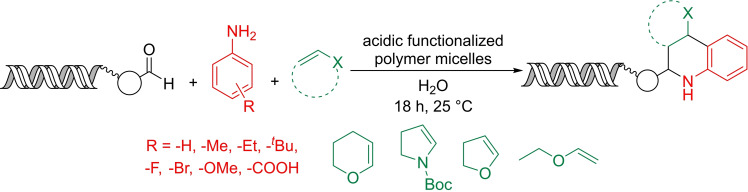
Application of acidic functionalized block copolymer as micelle forming agent and catalyst in Povarov reaction.

The Povarov reaction is a three‐component transformation which affords tetrahydroisoquinoline derivatives (Scheme 10). Under the optimized conditions a scope of eight aldehydes (conjugated to single stranded DNA through a linker), 23 anilines, and four electron‐rich olefins were investigated including the synthesis of two mock libraries each containing five members. DNA depurination was not observed under optimal conditions. Control experiments showed that the reaction proceeded with similar efficiency whether a 12‐mer or 21‐mer ssDNA was used. When a 69‐mer ssDNA was incubated with the copolymer catalyst under the reaction conditions and then subjected to PCR amplification the results were comparable to a control experiment performed without the catalyst. However, PCR amplification followed by sequencing of the amplified DNA was not reported.

In contrast to the Povarov reaction, Boc‐removal was accompanied by severe depurination. At first, it might seem contradictory that using the same catalyst the Povarov reaction proceeded without any DNA damage. However, the authors found that the addition of aniline or pyridine can mitigate depurination due to the buffering effect of the nitrogen containing bases.

### 2.4 Transfer hydrogenolysis and hydrogenation

**Scheme 11:**
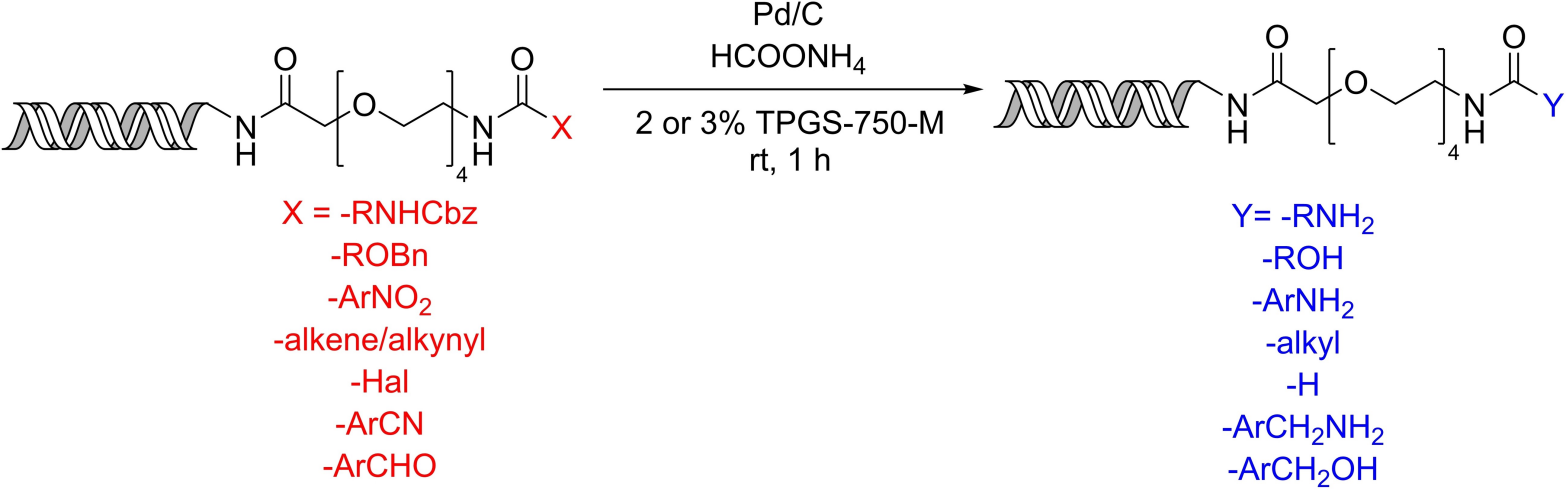
Micellar transfer hydrogenation and hydrogenolysis of DNA‐tagged substrates.

The combination of palladium on carbon and ammonium formate in the micellar environment of TPGS‐750‐M proved suitable for a variety of on‐DNA reduction reactions as shown by the Waring group (Scheme 11).^[33]^ Treatment of benzyl carbamates and benzyl ethers afforded the corresponding amines and alcohols, respectively. The reduction of nitro arenes, alkenes, alkynes, a benzonitrile, and benzaldehyde substrates was successfully performed under identical conditions using 2 or 3 % surfactant. The same method is suitable for the hydro‐dehalogenation of aryl halides.

When the reduction reaction was carried out without the TPGS‐750‐M, almost complete loss of DNA‐conjugated material was observed (<1 % recovery in all cases). However, control experiments indicated that the DNA chain remains intact under the applied optimal conditions.

To the best of our knowledge none of the above described DNA compatible micelle‐mediated reactions have been applied in library synthesis.

## 3 Well‐Established Reactions in Micellar Media: Unprecedented but Promising Candidates for DEL Synthesis

In this chapter we present a selection of micellar transformations which we believe to be prospective candidates for DEL synthesis. Our collection of catalytic and non‐catalytic reactions displays the power of micellar chemistry. Adoption of these reactions to DEL would offer access to diverse chemical libraries. Nevertheless, this is a subjective selection of prominent reaction types, and we aimed to discuss examples which have the potential to fulfil the criteria for DEL compatibility (Section 1.1).

### 3.1 Photoredox catalysis

Photoredox catalysis is a fairly new synthetic tool in organic chemistry,^[34]^ and offers a way to selectively generate radicals for non‐conventional coupling reactions utilizing a photocatalyst excited by visible light. Photoredox transformations are frequently carried out in organic solvents due to the hydrophobic nature of the applied photosensitizers. However, solubilization of photocatalysts in aqueous media can be increased with the aid of surfactants.

**Scheme 12:**
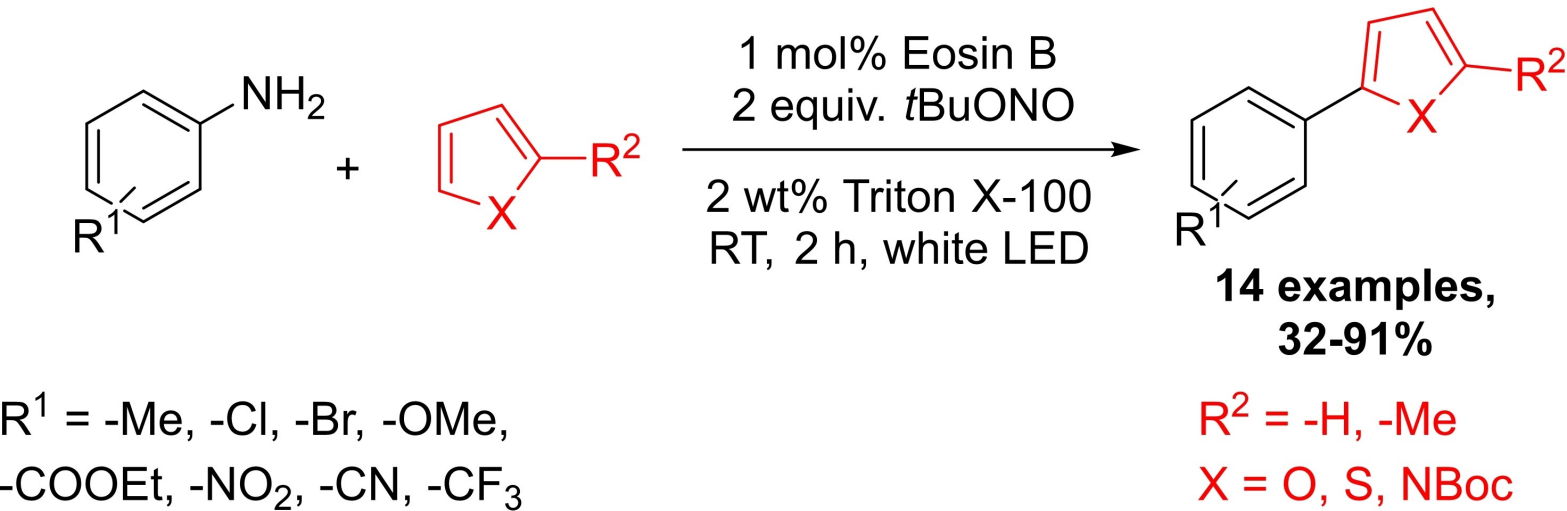
Photoredox coupling of heteroaromatics with diazonium ions generated in situ in micellar medium.

Cai and co‐workers worked out a photoredox coupling of heteroaromatics with in situ generated aryl diazonium ions using Eosin B organic dye as the photosensitizer (Scheme 12).^[35]^ Screening of photocatalysts and surfactants showed that the combination of 1 mol% Eosin B and Triton X‐100 worked the best. Without any surfactant, the model transformation proceeded only with 34 % efficiency. They showed the effectiveness of the developed method on 14 synthetic examples, which were isolated in 32–91 % yields. In general, anilines bearing electron‐withdrawing and ‐neutral substituents afforded higher yields (75–91 %) than those having electron‐donating groups. Steric effect did not significantly affect this transformation (*o*‐NO_2_: 84 %; *m*‐NO_2_: 81 %, *p*‐NO_2_: 86 %).

The same method was successfully applied for the *α*‐arylation of enol acetates (4 examples), a [4+2] benzannulation and for the synthesis of sulfides and selenides (7 examples). If this approach can be generalized for a broad range of substrate, it would certainly represent an attractive method for DEL, nonetheless there are some concerns: the nitrite reagent may oxidize purine bases of DNA and may react with the exocyclic amines present as well.

Giedyk and co‐workers developed a Minisci‐type micellar photoredox coupling using iridium based photocatalyst, CBr_4_ as the cocatalyst and SDS as the surfactant.^[36]^ This method was successfully applied for the C−H alkylation of caffeine's imidazole ring. One might wonder whether the structurally similar adenine and guanine nucleobases would be accessible for akin, yet undesired, C−H functionalization during a DEL application.

### 3.2 Palladium‐catalyzed carbon‐carbon cross‐couplings

Palladium catalyzed carbon‐carbon bond formation reactions are without a doubt among the most influential reaction types of the last decades.^[37]^ They offer a broad variety of transformations and became extensively applied in academic and industrial research and development, and even in production settings. Palladium‐catalyzed cross‐coupling reactions have been widely investigated for the synthesis of DNA‐tagged molecules, however, the demanding reaction conditions often lead to DNA damage.^[24]^ As discussed earlier, DEL compatible micellar Suzuki and Buchwald–Hartwig conditions have been developed by the Waring group.[^25^, ^28^] Below we discuss the state of the art of other types of prominent cross‐couplings under micellar conditions.

**Scheme 13:**
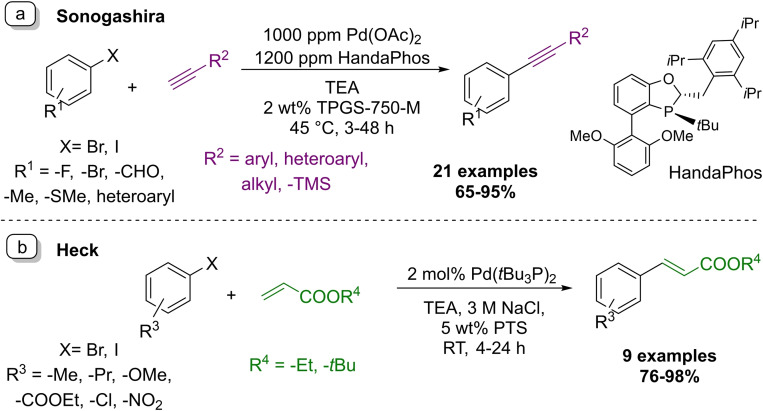
Examples of micellar palladium‐catalyzed cross‐coupling transformation developed by the Lipshutz research group.

The Lipshutz group developed a copper free micellar Sonogashira coupling using Pd(OAc)_2_/HandaPhos catalyst system (Scheme 13a).^[38]^ HandaPhos was designed specifically as a match for micellar catalysis, where lipophilicity becomes an impactful parameter. The use of HandaPhos allowed the authors to lower the transition metal concentration to ppm level.^[39]^ In this transformation aryl iodides proved to be more reactive than bromides, and gave the desired products at room temperature in 75–93 % yield range. The designed method was utilized in gram scale synthesis and recycling studies were carried out successfully. The surfactant medium could be used up to four times without significant change in preparative yields.

In 2011, a micellar Heck reaction between acrylic acid esters and aryl bromide and iodide derivatives was published (Scheme 13b).^[40]^ The method uses tri‐*tert*‐butyl phosphine palladium complex as the catalyst, with 5 wt% PTS solution as the reaction medium. Addition of NaCl led to accelerated reaction rates leading to shorter reaction times and allowed the transformation to take place at room temperature. Substrates functionalized in the *ortho* position offered slightly lower yield (76–82 %) than *meta*‐ and *para*‐substituted aryl bromides (86–98 %).

More recently, Lipshutz et al. utilized heterogenous iron/palladium nanoparticles as precatalysts for a micellar Mizoroki‐Heck‐reaction.^[41]^ The exposure of the nanoparticles to water initiated a morphology change, which allowed the active nanocatalyst to form in situ. The effectiveness of this technique was demonstrated on 26 synthetic examples from 82 % to quantitative yields.

**Scheme 14:**
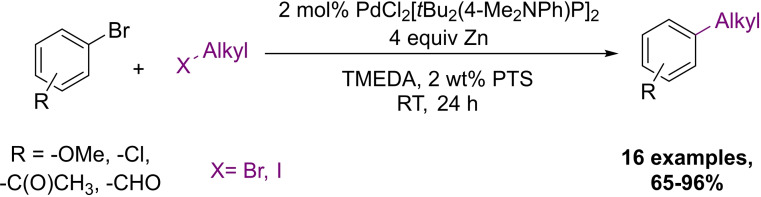
Palladium‐catalyzed Negishi coupling of alkyl zinc reagents generated in situ under micellar conditions

Transformations involving alkyl zinc reagents are rarely studied in protic media. With micellar solution Lipshutz and co‐workers reported the first aqueous Negishi‐type reaction in 2009 (Scheme 14).^[42]^ They generated the organometallic alkyl‐zinc coupling agent in situ by reacting fresh zinc powder with alkyl iodides and bromides in 2 wt% PTS solution.

Broad substrate scope, good functional group tolerance (even ketones and aldehydes remained intact under these conditions) and moderate‐to‐excellent yields were observed. The transformation has some steric dependency, while the *ortho* methoxy derivative were prepared only in 65 % yield, the *meta* variant could be isolated in 90 %. Control experiments confirmed the enabling role of the surfactant, as in its absence the reaction times became longer, and the conversion only reached 30 %.

### 3.3 C−H activation reactions

C−H activations allow for late‐stage functionalization without the need for lengthy functional group manipulations.^[43]^ However, the efficiency and selectivity of these reactions are often problematic and utilization of a directing group might be required.^[44]^ In respect to DEL, C−H functionalization holds great promise for diversification through atypical reaction handles and for the utilization of otherwise inaccessible building blocks.[^5a^, ^13^]

**Scheme 15:**
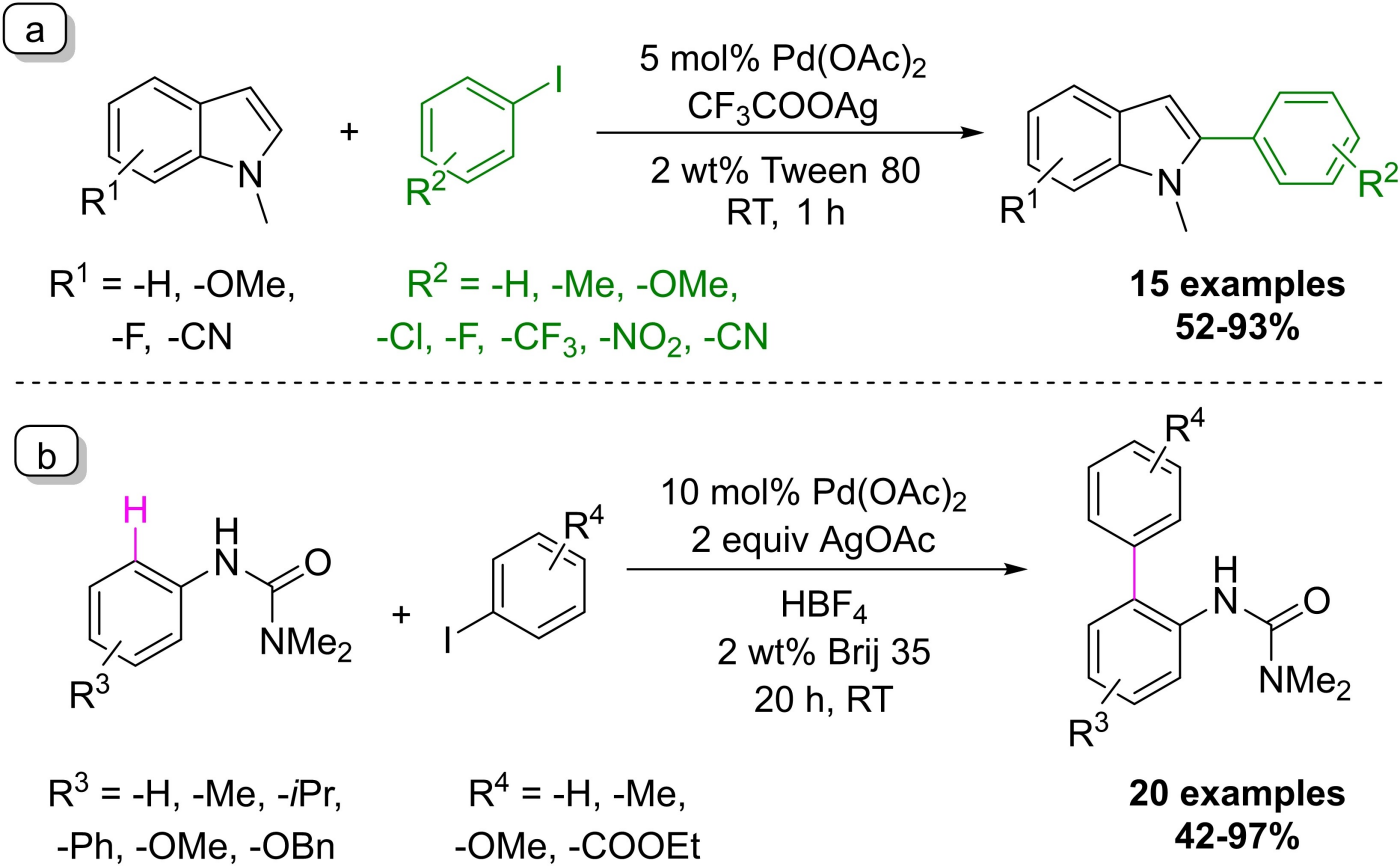
Examples of C−H activation performed under micellar conditions.

Shao and co‐workers published a micellar C−H activation transformation, a C(2)‐selective arylation of indoles using palladium catalyst and CF_3_COOAg as an additive (Scheme 15a).^[45]^ The coupling works efficiently at room temperature with only one hour of reaction time using aryl iodides as coupling partners in Tween 80 solution. However, without the addition of the surfactant, the efficiency of the transformation drops significantly, thus the Tween 80 is essential for the successful coupling. The performance of the developed method was demonstrated on 15 indole derivatives with 52–93 % isolated yields. Lower yields were observed when *ortho*‐substituted iodobenzenes were applied into the reaction. In addition, there was no C(3)‐arylation product observed in all tested substrates even for the steric hindered ones. They also extended the scope of the transformation for benzofuran and benzothiophene derivatives as well, however generally these substrates needed more reaction time (24–48 h). Similar to the Minisci‐type photoredox reaction, one might expect selectivity issues due to potential reaction of DNA purine bases at the C(8)‐position.

The Lipshutz group also developed a palladium‐catalyzed directed C−H activation transformation using aryl ureas and aryl iodides as substrates at room temperature (Scheme 15b).^[46]^ They utilized AgOAc as the additive and Brij 35 was found as the optimal surfactant of choice. Various directing groups were examined and only aromatic ureas reacted smoothly at room temperature. The effectiveness of the method was demonstrated on 20 synthetic examples, only sterically hindered aryl iodides were not reactive under these conditions.

### 3.4 Olefin metathesis

Olefin metathesis is a unique carbon skeleton redistribution reaction in which unsaturated carbon‐carbon bonds are rearranged in the presence of metal carbene complexes.^[47]^

**Scheme 16:**
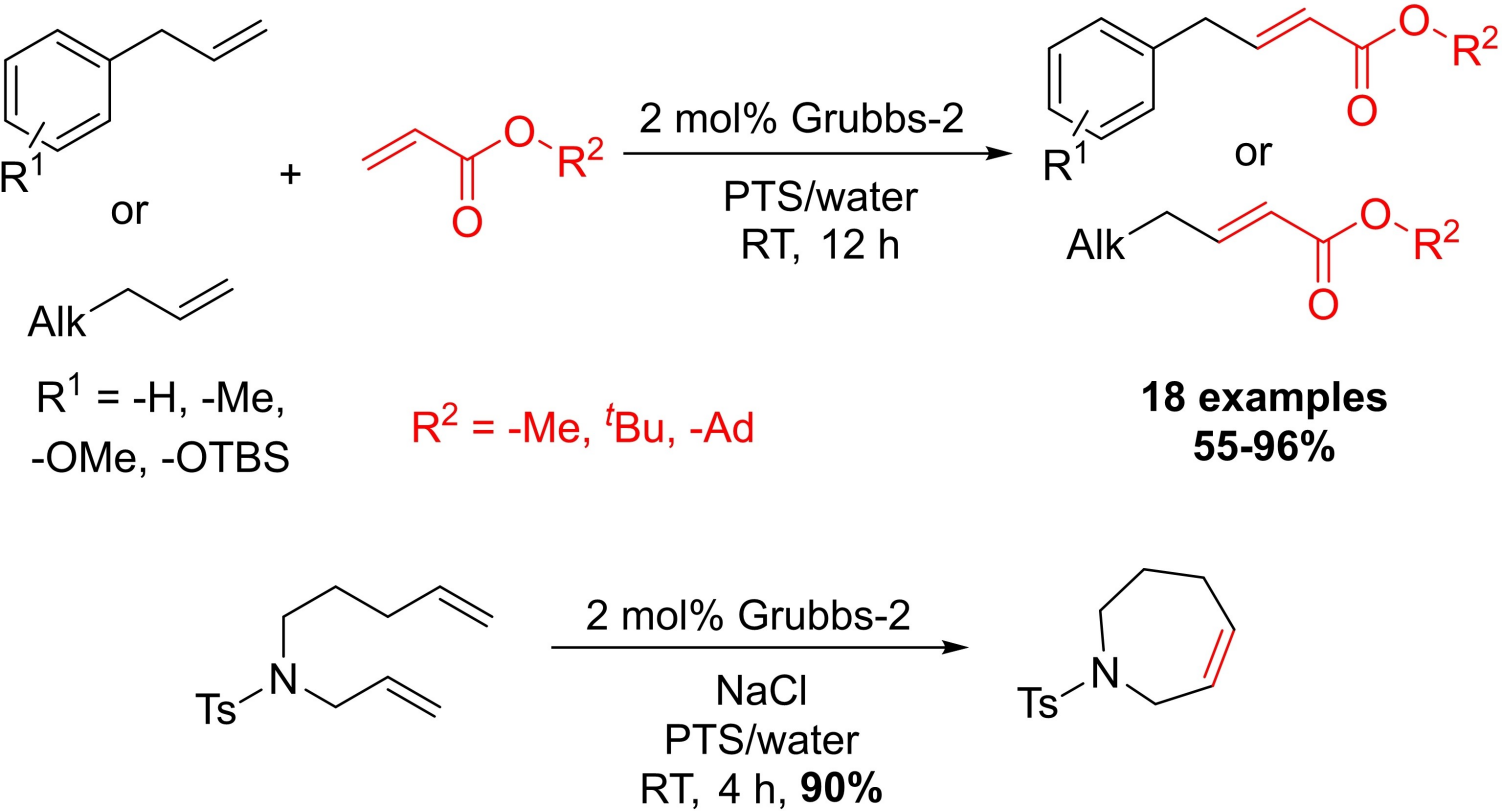
Olefin metathesis reactions carried out in micellar media by the Lipshutz research group.

In 2008, Lipshutz et al. published the first unsymmetrical olefin cross‐metathesis in water involving water‐insoluble substrates at ambient temperatures (Scheme 16).^[48]^ PTS was found as the best surfactant for this transformation in 2.5 wt% concentration, other non‐ionic surfactants produced more homocoupling side products lowering the selectivity, and without any amphiphiles the model reaction proceeded only with 62 % efficiency. A particularly noteworthy feature of this process is the comparable *E*/*Z* ratios to those observed in organic media. They also effectively implemented surfactant solutions in ring closing metathesis reactions, so the same conditions can be applied to cross‐metathesis as well as ring closing reactions.

### 3.5 Carbon‐heteroatom couplings: Buchwald–Hartwig reaction

The transition‐metal‐catalyzed carbon‐nitrogen bond formation reactions are also powerful tools to access aromatic amines,^[49]^ and the DEL community have showed significant interest in this transformation as well.^[50]^ Very recently even a micellar example was published,^[28]^ nevertheless, the scope of on‐DNA transition‐metal catalyzed C−N coupling reactions is still limited.

**Scheme 17:**
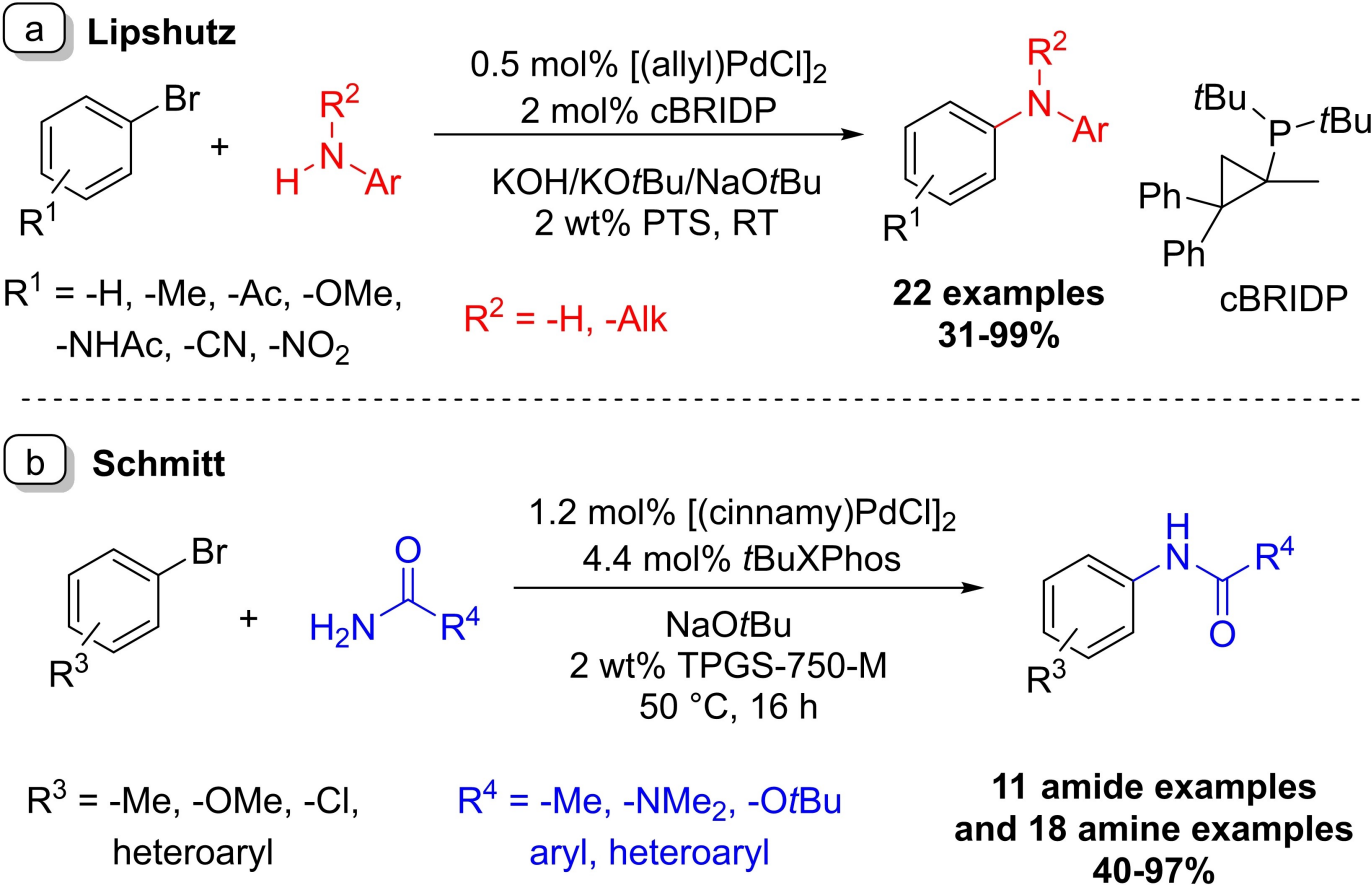
Buchwald–Hartwig couplings in surfactant solutions.

In 2009 the Lipshutz group published their aqueous Buchwald–Hartwig coupling reaction between bromobenzene derivatives and aromatic amines (Scheme 17a).^[51]^ Their surfactant choice for this transformation was PTS, and it was found that the most effective ligand for this coupling is Takasago's cBRIDP ligand.^[52]^ The efficiency of the developed method was demonstrated on 22 examples. Lower yields were observed when ortho‐substituted aryl bromides were used, on the other hand an aniline, bearing substituents in the same positions appeared to be more forgiving. The developed micellar technique was directly compared to a known organic synthetic method. The original synthesis used DME as the solvent at 100 °C with 20 h of reaction time while providing only 56 % yield. Under the micellar catalytic conditions, the reaction took place at room temperature and provided 84 % product in 3 h.

Schmitt and co‐worker's research was based on the previously shown Lipshutz transformation (Scheme 17b).^[54]^ The cBRIDP ligand limited the substrate scope of the original reaction so their aim was to broaden the applicability of the coupling. During their reaction development 15 different phosphine ligands were tested, and *t*BuXPhos was found to be the most effective paired with [(cinnamyl)PdCl]_2_ precatalyst. The method worked efficiently even when benzamides were used as substrates. Under the conditions developed all amides gave good conversion except for the highly soluble acetamide, which needed to be applied in excess.

A more recent micellar example from the Lipshutz group also utilized the *t*BuXPhos ligand and [(cinnamyl)Pd‐complex catalytic system for the coupling of aryl‐bromides with a wide variety of amine derivatives.^[53]^

**Scheme 18:**
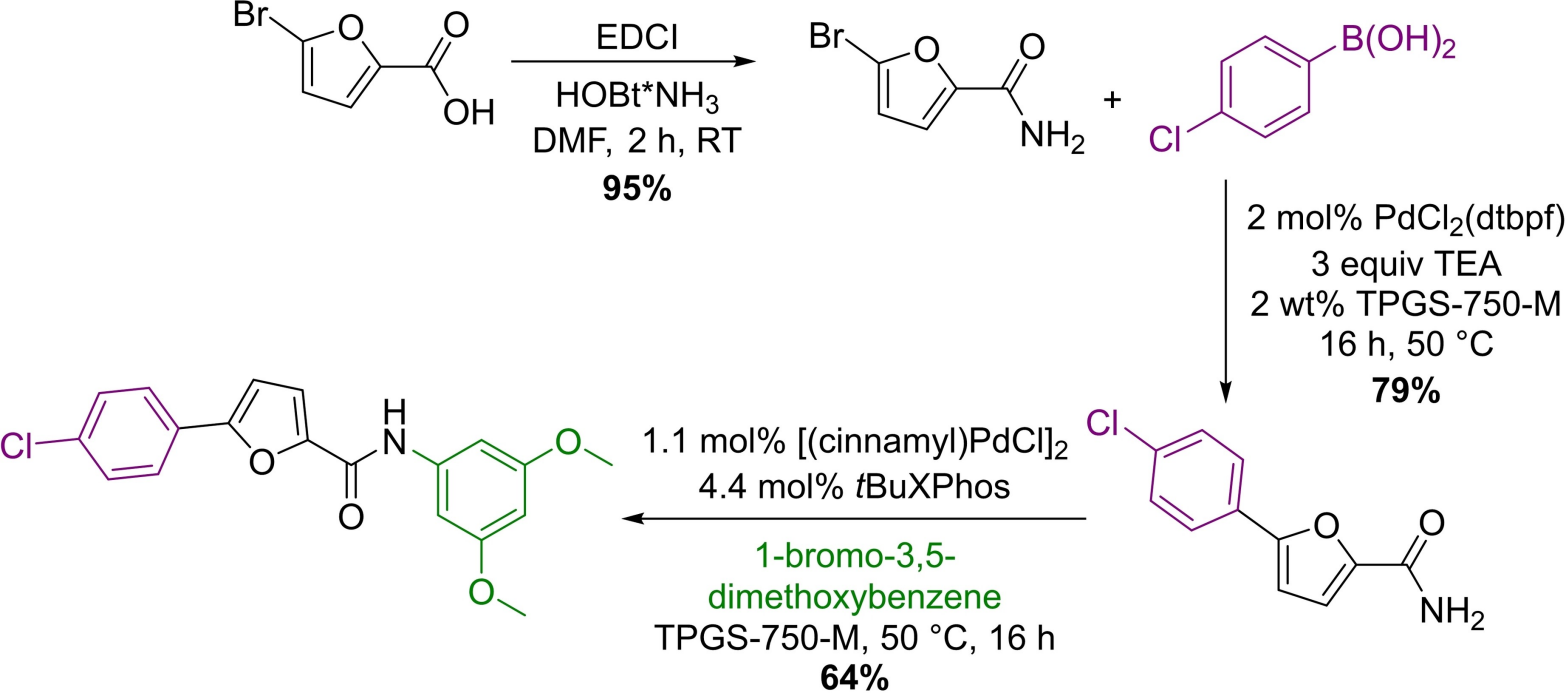
Three‐step synthesis of 5‐aryl‐2‐furfuramide in micellar Suzuki‐ and Buchwald–Hartwig reactions

The same methodology was applied in the three‐step synthesis of 5‐aryl‐2‐furfuramide, a potent and selective blocker of the NaV_1.8_ sodium channel.^[55]^ Easily available 5‐bromo‐2‐furfuramide was coupled with 4‐chloro‐phenylboronic acid under micellar catalytic conditions. After isolation, a subsequent Buchwald–Hartwig coupling reaction with 1‐bromo‐3,5‐dimethyoxybenzene provided the desired product with 48 % overall yield, which was comparable with the process reported in the literature (Scheme 18).

### 3.6 Nucleophilic aromatic substitutions

Aromatic nucleophilic substitutions are potent synthetic tools for the formation carbon‐heteroatom bonds. This methodology is widely used in pharmaceutical chemistry due to its transition metal free approach. Application of S_N_Ar in DEL have been described.^[56]^

**Scheme 19:**
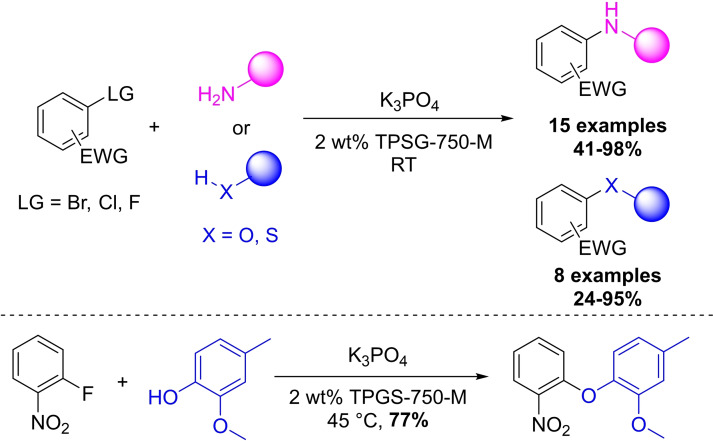
Micellar aromatic nucleophilic substitution by using nitrogen, oxygen and sulfur nucleophiles, as well as the synthesis of antibacterial FabI inhibitor.

The Lipshutz group used aromatic halogen compounds (usually bromides, chlorides and fluorides) with an electron withdrawing group, like nitro group, or heteroaromatic compounds as substrates in nucleophilic aromatic substitutions with 2 wt% TPGS‐750‐M solution as the reaction medium (Scheme 19).^[57]^

They demonstrated the effectiveness of their method on 22 synthetic examples, nitrogen‐based nucleophiles reacted smoothly using a 1 : 1 reagent substrate ratio, while oxygen‐ and sulfur‐based nucleophiles were less reactive, and mild heating (45 °C) was needed in most cases to complete the transformations. Benzylic alcohols and phenol derivatives offered high yields however alkanols were more problematic in terms of reaction conditions and efficiency, requiring the presence of 10 mol% DMAP and still gave only ∼24 % yield. They also conducted side by side comparison experiments between the aqueous surfactant solution and DMF and they found that using micellar conditions leads to comparable reaction rates. The preparation of three known pharmaceutical structures were also carried out successfully, one of them is an antibacterial FabI inhibitor.

## 4 Summary and Outlook

Micellar chemistry has become an elaborated technique in the synthesis of small organic molecules offering a broad range of transformations to choose from. Micellar aqueous solutions can be used as alternative reaction media to organic solvents, as micelles can enhance the reaction rate at mild temperatures, acting as nanoreactors effectively solubilizing highly hydrophobic reagents and catalysts. In addition, the applied surfactant solution can be recycled and reused several times, thus providing advantageous economic and environmental benefits.

Micellar reactions usually proceed under mild conditions (<60 °C) and offer the compartmentalization of different reagents based on their hydrophilic/phobic nature in aqueous media. These properties seem to render micellar chemistry ideal for DEL applications. Very recently, the teams of Weberskirch and Brunschweiger, and Waring have shown that, in fact, micellar chemistry can provide new avenues in DEL technology. It was demonstrated that DNA conjugates can tolerate reactions under acidic conditions when the acidic sites are located inside micelles. Of similar importance, DNA‐compatible, palladium‐catalyzed couplings, which often lead to the degradation of DNA, were successfully developed by using micellar catalysis. Nevertheless, these examples also showed that DNA‐encoded micellar chemistry has its own challenges and is still in its infancy. All of the published examples featured a substrate scope that is below the practical requirements of DEL, and to the best of our knowledge, micellar chemistry has not been applied in library synthesis.^[58]^ Furthermore, the choice of surfactant and headpiece linker is not straightforward, and might require additional optimization. DNA micelles have been applied for DNA‐templated organic reactions.^[59]^ One might wonder whether DNA conjugates would be applicable to form micellar nanoreactors and at the same time serve as barcodes for DEL.

Overall, we believe that micellar chemistry represents a promising approach for DEL synthesis and significant progress can be expected in the next couple of years. We hope that the current contribution will facilitate the evolution of micellar DNA‐encoded library chemistry.

## Conflict of interest

The authors declare no conflict of interest.

5

## Biographical Information

*Réka Adamik received her B.Sc. from Eötvös Loránd University, Budapest in 2016 and performed M.Sc. studies at the Budapest University of Technology and Economics. She joined Zoltán Novák's research group in 2015, and since 2018 she has been working on her PhD. research under his guidance*.

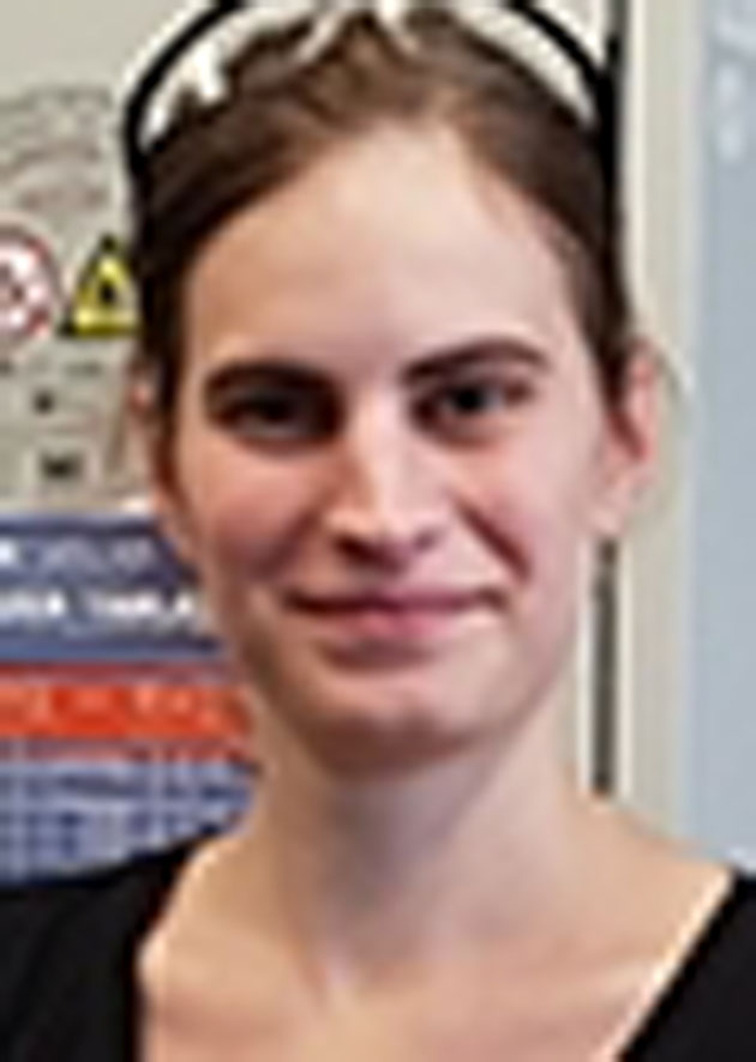

## Biographical Information

*Gellért Sipos received his MSc in chemistry from Eötvös Loránd University (ELTE) in 2011 and his PhD from the University of Western Australia in 2017. Following postdoctoral research at ELTE, he joined ComInnex and ThalesNano. Currently, his research focuses on flow chemistry, photochemistry, and DNA‐encoded library technology*.

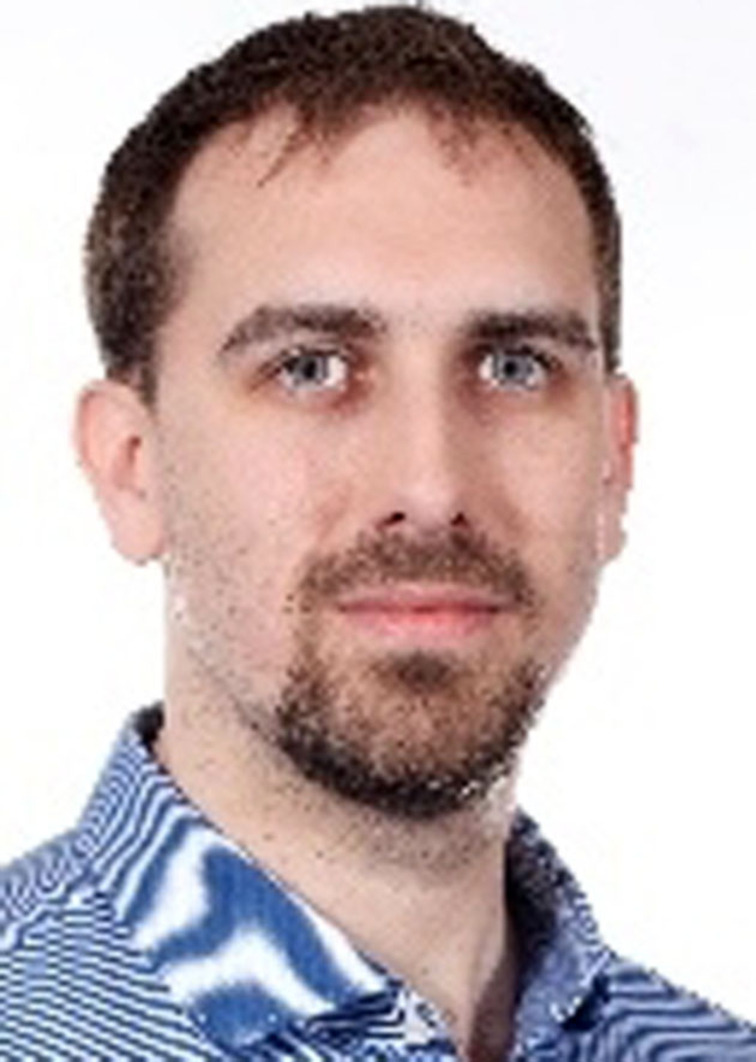

## Biographical Information

*Balázs Buchholcz obtained his M.Sc (2015) and Ph.D. (2018) in nanotechnology and materials science from the University of Szeged, Hungary. He joined the board of Innostudio in 2019. He manages the nanotechnology and space chemistry laboratory, focusing on the application and development of state‐of‐the‐art technology solutions*.

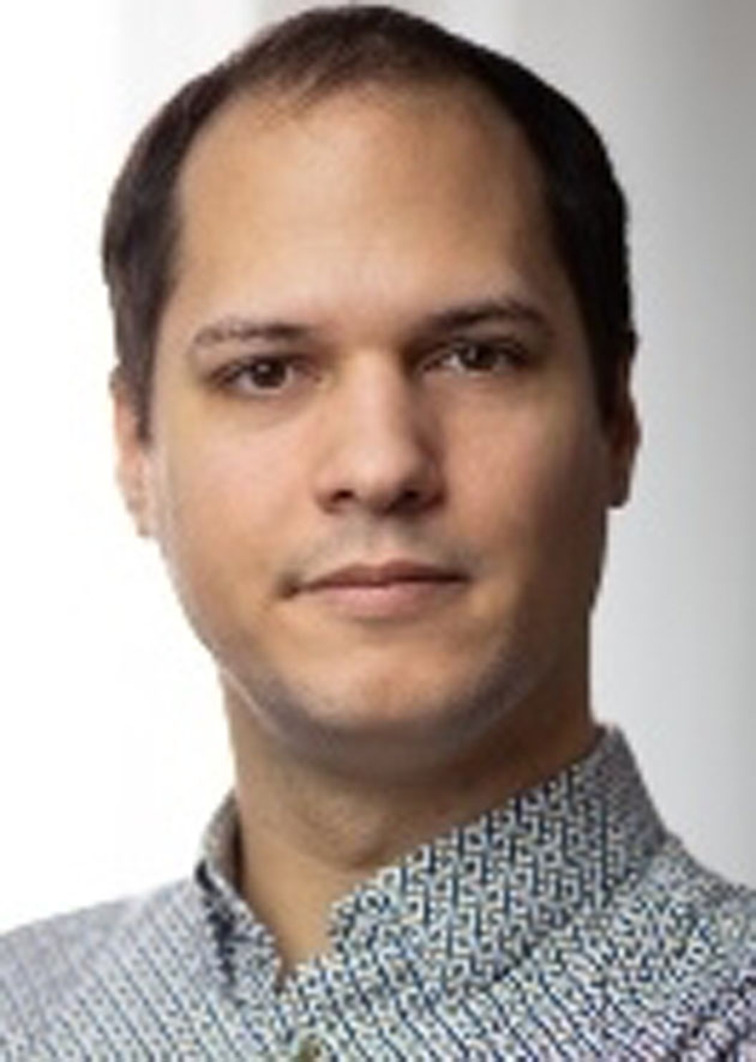

## Biographical Information

*Zoltán Novák performed his M.Sc. and PhD. studies at Eötvös University (ELTE) with Professor András Kotschy, obtaining his degrees in 1999 and 2004 respectively. In 2004, he joined the group of Professor Brian M. Stoltz at the California Institute of Technology as a postdoctoral researcher. After returning to Hungary in 2005, he continued his research at ELTE, again in the group of Professor Kotschy. In 2007, he started his independent research career at the Department of Organic Chemistry, Institute of Chemistry at ELTE as an associate professor, becoming a full professor in 2020*.

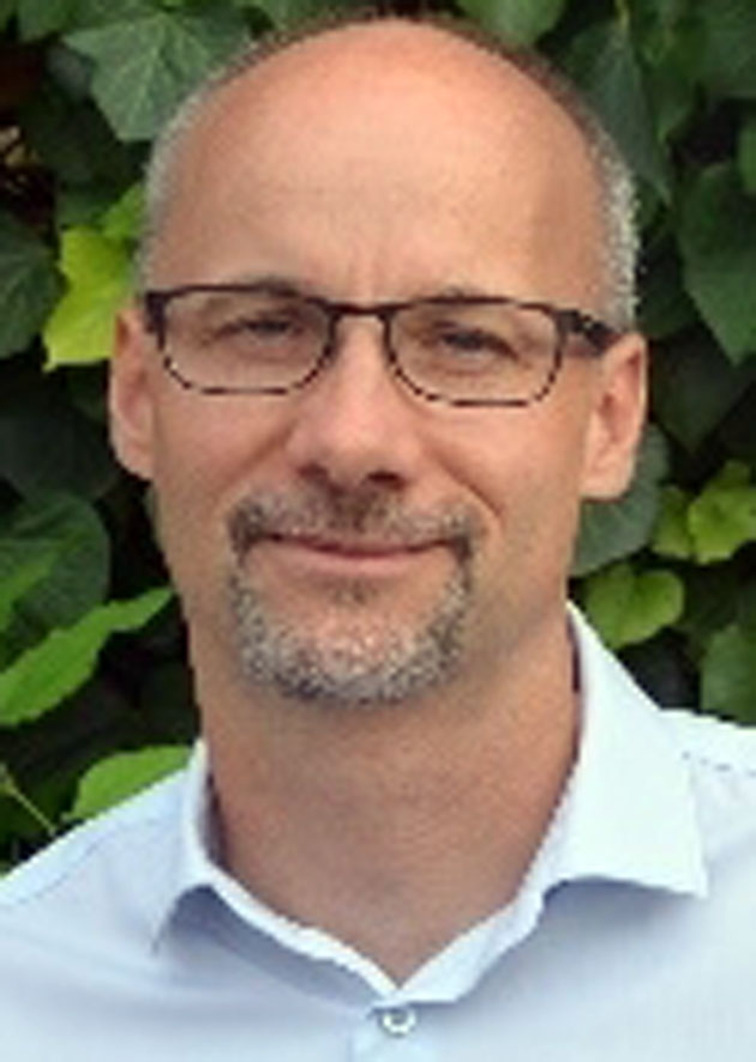

## Biographical Information

*Ferenc Darvas has a MS degree in medical chemistry, a BS in computer sciences, a degree in patent law, and a PhD in experimental biology. He has taught in Hungary, Spain, Austria, and the USA and is author of over 170 peer‐reviewed papers and five books. He has been involved in introducing microfluidics/flow chemistry methodologies for synthetizing drug candidates since the late 1990s, which led him to found ThalesNano, the inventor of H‐Cube®. Presently he is focusing on space chemistry research*.

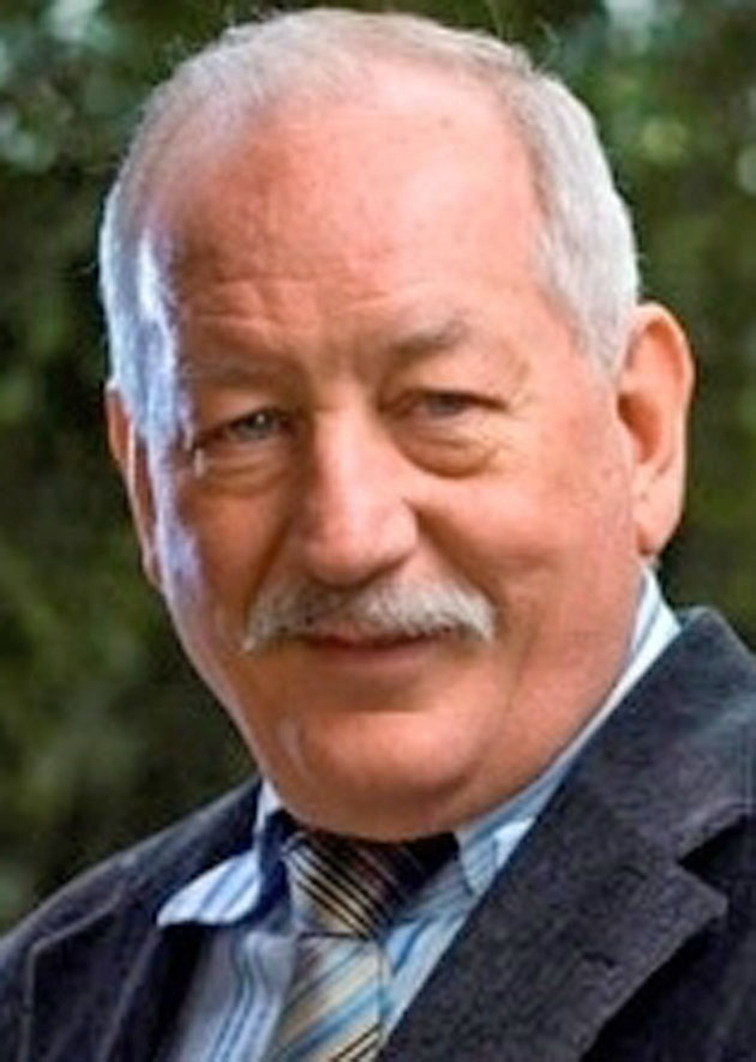

## Data Availability

Data sharing is not applicable to this article as no new data were created or analyzed in this study.
